# Introduction to the Special Issue on “Informing Longitudinal Studies on the Effects of Maternal Stress and Substance Use on Child Development: Planning for the HEALthy Brain and Child Development (HBCD) Study”

**DOI:** 10.1007/s42844-020-00022-6

**Published:** 2020-10-22

**Authors:** Chloe J. Jordan, Susan R. B. Weiss, Katia D. Howlett, Michelle P. Freund

**Affiliations:** National Institute on Drug Abuse, National Institutes of Health, 3WFN RM 09C71 MSC 6021, 301 North Stonestreet Ave, Bethesda, MD 20892 USA

**Keywords:** Brain, Development, Substance use, Environment, Exposure

## Abstract

The HEALthy Brain and Child Development (HBCD) study will establish a large cohort of pregnant women from regions of the country significantly affected by the opioid crisis and follow them and their children for at least 10 years. Findings from this cohort will help researchers understand normative childhood brain development as well as the long-term impact of prenatal and postnatal opioid and other drug and environmental exposures. The study will collect data on pregnancy and fetal development; infant and early childhood structural and functional brain imaging; anthropometrics; medical history; family history; biospecimens; and social, emotional, and cognitive development. Knowledge gained from this research will be critical to help predict and prevent some of the known effects of prenatal and postnatal exposure to certain drugs or environmental exposures, including risk for future substance use, mental disorders, and other behavioral and developmental problems. In this special issue, a subset of investigators that received funding for planning grants for the HBCD study provide careful guidelines and frameworks for study design, recruitment and retention of vulnerable populations, culturally sensitive practices, and biospecimen and neurodevelopmental assessment recommendations gathered in feasibility studies that will help inform the full HBCD study planned to begin recruitment in 2022.

## Background

The brain undergoes rapid development prenatally, through early childhood and into adolescence. Brain volume doubles in size in the first year of life (Gilmore et al., 2007; Gilmore, Knickmeyer, & Gao, 2018), and cortical surface area expands by 76% (Gilmore et al., 2018; Li et al., 2013). These rapid periods of growth support many aspects of cognitive maturation and also represent highly vulnerable periods when a variety of environmental and other exposures can have immediate and long-term impacts on health and neurodevelopmental outcomes. Exposure to environmental teratogens and toxins, nutritional deficiencies, stress, home and neighborhood factors, healthcare inequities, racial and ethnic disparities and systemic racism, viruses, and other pathogens can influence growth and brain development in myriad ways.

Increases in substance use over the past decade have heightened the urgency for understanding the complex ways in which environmental and other exposures during pregnancy affect child outcomes. The current opioid crisis affects the nation broadly across socio-demographic groups, with the number of women presenting with opioid use disorder (OUD) at labor and delivery increasing fourfold over a 15-year period from 1999 to 2014 (Haight, Ko, Tong, Bohm, & Callaghan, 2018). The number of infants born with neonatal opioid withdrawal syndrome (NOWS) increased fivefold between 2004 and 2014 (Winkelman, Villapiano, Kozhimannil, Davis, & Patrick, 2018). Recent estimates indicate that approximately 8.8/1000 in-hospital births are affected by NOWS or neonatal abstinence syndrome (NAS; Leech, Cooper, McNeer, Scott, & Patrick, 2020). A growing body of evidence, albeit in relatively small sample sizes, indicates that early exposure to opioids and other potentially harmful substances, both pre- and perinatally, is linked to a number of adverse outcomes. These adverse outcomes include low birth weight and preterm birth, altered brain structure and function early in life, and behavioral problems in childhood and adolescence, including altered cognitive function, attention-deficit/hyperactivity disorder (ADHD), conduct disorder, early drug use, and anxiety (Boggess & Risher, 2020; Nygaard, Slinning, Moe, Fjell, & Walhovd, 2020; Yeoh et al., 2019). Medications to treat OUD in pregnant women may improve pregnancy and child outcomes with potential gains projecting into adulthood, but the long-term effects of those medications also remains largely unknown.

The relationship between prenatal opioid exposure, the occurrence of NOWS/NAS, and long-term outcomes can be further augmented or mitigated by economic conditions, healthcare availability, and other environmental factors (Patrick et al., 2019). In addition, substance exposures during pregnancy are not limited to opioids. Cannabis, tobacco/nicotine, and alcohol are also used alone and in combination during pregnancy. Reports of cannabis use during pregnancy nearly doubled between 2002 and 2017 (Volkow, Han, Compton, & McCance-Katz, 2019; although these rates were lower in subsequent years; McCance-Katz, 2018), and reports of rising cocaine and methamphetamine use over the last two decades in pregnant women from some geographical areas, with corresponding increases in low birth weight, preterm delivery, and maternal morbidity, continue to accumulate (Smid, Metz, & Gordon, 2019). The COVID-19 pandemic has added another layer of stress to many individuals and families, and the virus itself may directly or indirectly affect neurodevelopmental outcomes. However, the relative paucity of research on normative brain development from birth and through childhood from a large, diverse cohort has limited our ability to fully understand how experiences during early periods of growth, including exposure to substances, impact individual developmental trajectories.

## The HEALthy Brain and Child Development Study

Establishing causal links between early exposure to substances or other potential adversities and future health and behavioral consequences is complex and will require a large prospective study of children beginning prenatally and followed into childhood, with detailed characterization of their brain development using neuroimaging and physiological tools and genetic, social, behavioral, and other biological/environmental contextual assessments. To understand how substance exposures and other environmental factors, alone and in combination, interact with genetics and other biological influences to affect a child’s mental and physical health trajectory, the NIH Helping to End Addiction Long-term^SM^ (HEAL) Initiative and the National Institute on Drug Abuse (NIDA), the National Institute of Neurological Disorders and Stroke (NINDS), the National Institute of Mental Health (NIMH), the National Institute on Alcohol Abuse and Alcoholism (NIAAA), the Eunice Kennedy Shriver Institute of Child Health and Human Development (NICHD), the Office of Behavioral and Social Sciences Research (OBSSR), the Office of Research on Women’s Health (ORWH), the National Institute of Environmental Health Sciences (NIEHS), and the National Institute on Minority Health and Health Disparities (NIMHD) intend to jointly fund the HEALthy Brain and Child Development (HBCD) study.

By establishing a nationwide, multi-site, multi-modal, longitudinal cohort study that prospectively examines the brain and behavioral development from birth through childhood, the HBCD study will provide multifaceted and detailed information on neurodevelopmental trajectories and the long-term impact of high-risk environments, including substance exposures. The HBCD study will enroll a large cohort of pregnant women, who, along with their children, will be followed for up to ten years. This study will include diverse populations to determine normal variability in development and factors that may disrupt it or build resilience across a variety of contexts. The study will also be of sufficient duration and scope to detect potential impact that may manifest as the child transitions through childhood. Accordingly, while a majority of the cohort is expected to be recruited from the general population, a subset will include pregnant women whose babies were exposed pre- or perinatally to prescription and/or illicit opioids, marijuana, stimulants, alcohol, and tobacco/nicotine, as well as women from comparable high-risk environments who did not use substances during pregnancy. Advances in neuroimaging, bioinformatics, and genetic technologies (among others) will enable the collection of multi-modal data that will be made available on an ongoing basis to the wider research community for hypothesis testing, timely data analyses, and as a long-term resource for studying neurodevelopment and other health outcomes. A deep, nuanced understanding of factors that affect a child’s health, brain, and behavioral development is expected to emerge from this study. This will be an essential first step toward designing policies and interventions that promote well-being and resilience in all children.

## Planning for the HBCD Study

**Fig. 1:**
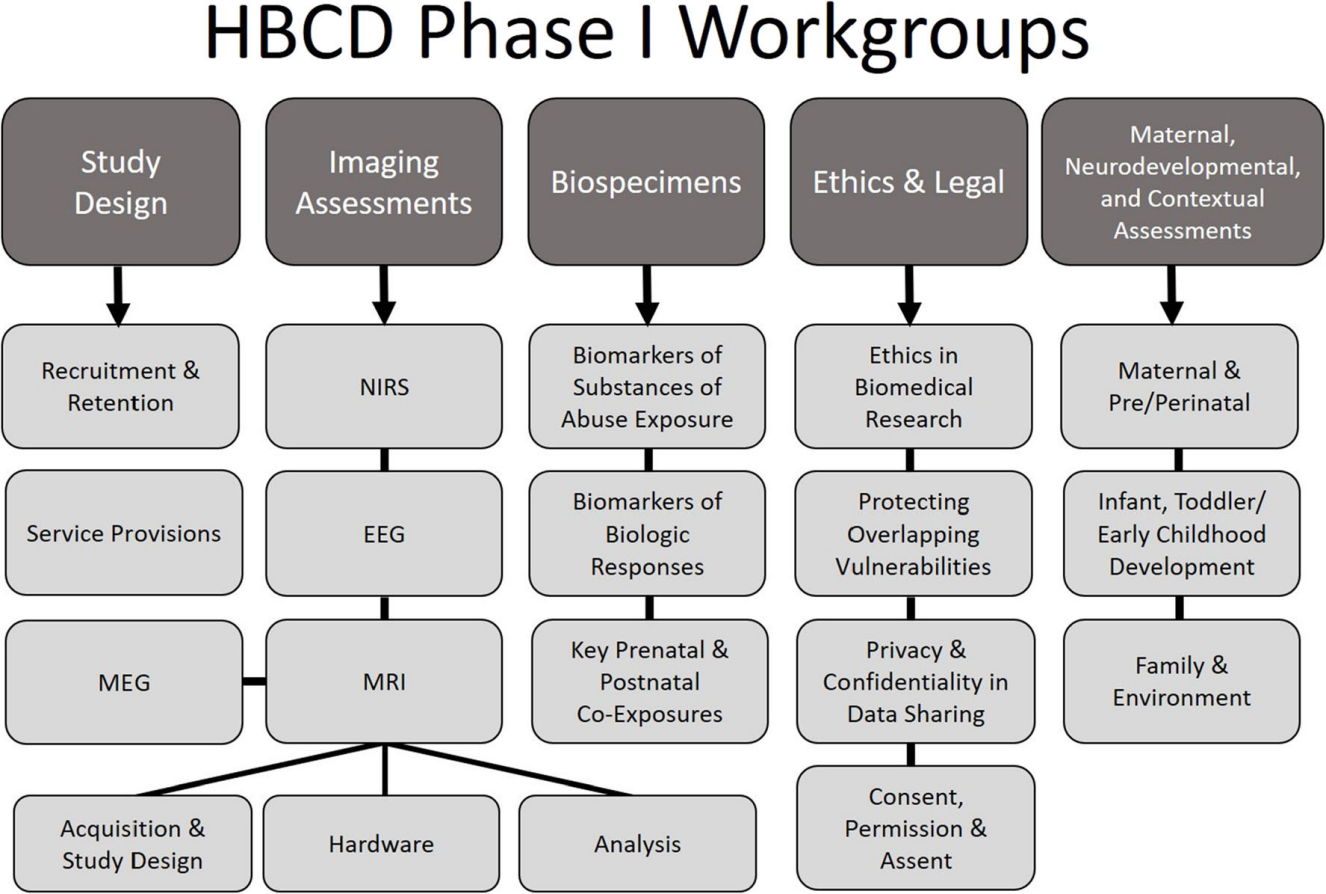
A diagram illustrating the organization of the five major nationwide working groups and relevant subgroups formed during phase I of the HBCD study

In recognition of the complexities faced by a large-scale longitudinal cohort study focusing on pregnant women with and without substance use and from high-risk backgrounds, as well as the challenges of neuroimaging, biospecimen collection, and frequent assessments in infants and children, a planning phase for the HBCD study was initiated in Fall 2018. The planning phase involved the funding of 29 sites across the USA, which sought to establish feasibility of neuroimaging and neurophysiological protocols in infants and children, determine effective recruitment and retention strategies for pregnant women and their infants, understand the ethical and legal complexities of recruiting pregnant women with substance use, and more. As part of these efforts, investigators, staff, and colleagues formed five nationwide working groups with more than 18 subgroups in key domains (Fig. 1), including neuroimaging and neurophysiology, study design, ethical and legal considerations, biospecimen collection, and maternal, neurodevelopmental, and contextual assessments. These workgroups met weekly or biweekly to discuss progress, identify challenges and brainstorm solutions, and address key questions to help inform the funding opportunity announcements and protocols for the full-scale HBCD study. Summary documents from these workgroups and meeting reports are made available on the HEAL website (https://heal.nih.gov/research/infants-and-children/healthy-brain). The HBCD study funding announcements are open to all investigators, regardless of whether they were a part of the feasibility phase.

## Overview of the Special Issue

The papers presented in this special issue of *Adversity and Resilience* represent just some of the valuable work done by the phase I HBCD study investigators and their workgroups that will guide the full HBCD study, as well as inform other research initiatives of similar goals and scope. Beginning with a historical perspective, in their paper “50 years of research on prenatal substances: lessons learned for the opioid epidemic,” Singer and colleagues highlight ways in which research from prior decades on prenatal drug and alcohol exposure can inform studies today. A historical perspective is particularly critical in light of the inaccurate conclusions and stigma that were promulgated throughout the 1980s and 1990s about so-called crack babies. These early studies highlight the importance of contextualizing research findings and including proper control groups, to avoid drawing premature and misleading conclusions that can stigmatize and damage families. Another lesson of the past is that substance or other environmental exposures may not result in visible physical defects but may rather manifest as unseen impacts on neurodevelopment. The impact of prenatal substance exposure on neurodevelopment is mediated by the timing of exposure during development, the dose, frequency, and pattern of exposure (such as binge consumption), as well as other environmental and biological factors that can convey additional developmental consequences. Studies on prenatal exposures are further complicated by the fact that illicit drugs are often adulterated, and polysubstance use is common. To account for these factors to the best extent possible, the HBCD study must employ multi-modal assessments of early substance use and exposure, including interviews as well as biosampling. Singer and colleagues caution that biomarkers for substance use or exposure are limited and cannot accurately assess first-trimester use when a great deal of brain development occurs and so should be combined with self-report. Methods like the Timeline Followback Assessment that focus on anchoring participant’s memories in key salient dates to enhance recall are recommended. Research teams must cultivate a rapport, assure confidentiality, and establish trust with participants who may be hesitant to share information about substance use due to fear of repercussions or stigmatization.

Beasley and colleagues take a deeper look at factors that can affect recruitment and retention in vulnerable populations, particularly pregnant women and those with substance use, in their paper “Understanding best practices in engaging pregnant and postpartum women at risk of substance use in longitudinal research studies.” The success of longitudinal studies on vulnerable populations critically depends upon understanding both barriers and motivators to study participation. In the past, barriers have included complex factors such as the severity of substance use, housing stability, legal systems, parenting, relationship, and employment challenges, and the need for intensive services. The authors stress that failure to manage these barriers can lead to a sample of convenience that is not representative of the target population, which directly opposes the goals of the HBCD study. Motivators to study participation may include understanding of the study benefits (both for the mother-infant dyad and from an altruistic perspective), as well as compensation, transportation, childcare, flexibility, relationship building with families, and demonstrating respect and compassion. The source and quality information about the study may also be a key factor in recruitment and retention, with social media and trusted locations such as childcare centers and clinics being valued, as well as education about the study procedures and protocol logistics for neuroimaging and biospecimen collection. The authors emphasize that leveraging these motivators and multi-modal methods for reaching out to participants can reduce barriers to participation and improve both recruitment and retention of diverse populations.

Croff and colleagues, in their paper “Early environmental exposures and contaminants: a design framework for biospecimen collection and analysis for a prospective national birth cohort” provides a comprehensive overview of the framework that will guide planning for biospecimen collection for the HBCD study. There is a complex interplay between pre- and postnatal factors that influence neurodevelopment, and studies like HBCD can critically begin to separate the teratogenic effects of substance exposure from other environmental adversities. The biospecimen working group began by reviewing protocols developed by other nationwide studies and repositories engaging in biospecimen collection, thus anchoring their efforts in pre-existing and well-validated work. Biomarkers were then selected as essential or recommended for collection based on developmental stage (focusing on prenatal through early childhood) and participant type (infant, mother, father), in four key domains: (1) substance use exposure, (2) other environmental exposures, (3) genomics, and (4) other biomarkers such as stress, inflammation, and the microbiome. The authors provide guiding principles for the classification of each potential specimen, with careful weighting of the scientific utility of the specimen vs. logistics and cultural sensitivities.

In their paper “Inclusion of Native Americans and Alaskan Natives in large national studies: ethical considerations and implications for biospecimen collection in the HE**A**Lthy Brain and Child Development Study,” Bakhireva and colleagues further explore the importance of inclusion of racial and ethnic minorities through culturally sensitive study practices, focusing on American Indian and Alaskan Native (AI/AN) communities. As a starting point, researchers must recognize the history of unethical, insensitive, exploitative and stigmatizing treatments of AI/AN peoples. This paper underscores forced migration, boarding schools, and attempted eradication of AI/AN culture as just some examples of the gross maltreatment of the past, resulting in a lack of trust that persists today. Done well, building sensitivity to ethical and cultural issues into research practice can further improve recruitment and retention and enhance the diversity of participants. However, if done improperly, studies may exacerbate existing stigmatization and mental health conditions within AI/AN communities. Bakhireva and colleagues encourage researchers to build relationships with AI/AN tribes and other appropriate entities, including representation of Urban Indians who may not live on reservation or tribal lands. By outlining guiding principles in creating partnerships with AI/AN communities and engaging in culturally respectful and meaningful study design and data management, this paper provides a valuable framework for researchers to reference in developing not only the HBCD study but also scientific protocols of any focus and scope.

In addition to biospecimen collection to determine substance and other environmental exposures, neurodevelopmental assessments will be vital in assessing infant and child outcomes and understanding risk vs. resilience to those exposures. Morris and colleagues, in their paper “Principles for guiding the selection of early childhood neurodevelopmental risk and resilience measures: HEALthy Brain and Child Development (HBCD) Study as an exemplar,” draw a framework for assessment selection to guide HBCD study investigators as well as researchers in other large-scale neurodevelopmental studies. This framework balances key considerations for assessment collection in terms of pragmatics, measurements, and harmony with other ongoing developmental studies and protocols while recognizing that assessment of developmental outcomes requires consideration of the child in the broader context of family, environment, and history. Additional priorities highlighted for assessment selection include availability in multiple languages, scalability and flexibility, utility to communities and stakeholders, and strength-based assessments of well-being and positive health. With these guiding principles in mind, the paper recommends measures in several key domains of relevance not only to HBCD but also to many neurodevelopmental studies, including socio-emotional development, developmental psychopathology, cognitive development and executive function, language development, and motor and physical development. The paper underscores that research in the past has been divided between developmental and clinical science. This division has hindered efforts to reconcile common neural pathways that may be contributing both to child temperament and the development of psychopathology. By adapting a trans-diagnostic approach in studies like HBCD, impending pathologies may be anticipated by earlier emerging traits such as internalizing and externalizing behaviors.

In summary, the papers presented in this Special Issue provide careful guidelines and frameworks for study design, recruitment, and retention of vulnerable populations, culturally sensitive practices, and biospecimen and neurodevelopmental assessment recommendations that may be valuable to both the forthcoming HBCD study and other neurodevelopmental research efforts. We hope that current and future research teams, as well as the broader neurodevelopmental science community, will find this special issue to be informative and inspiring in guiding future research endeavors, including the HBCD study.
